# Multiple Aerial Targets Re-Identification by 2D- and 3D- Kinematics-Based Matching

**DOI:** 10.3390/jimaging8020026

**Published:** 2022-01-28

**Authors:** Shao Xuan Seah, Yan Han Lau, Sutthiphong Srigrarom

**Affiliations:** 1 Department of Mechanical Engineering, Faculty of Engineering, National University of Singapore, 9 Engineering Drive 1, Singapore 117575, Singapore; seahshaoxuan@u.nus.edu; 2 Department of Electrical and Computer Engineering, Faculty of Engineering, National University of Singapore, 4 Engineering Drive 3, Singapore 117583, Singapore; yanhan.lau@u.nus.edu

**Keywords:** kinematics-based matching, multi-camera multi-target tracking (MCMTT), target re-identification, graph matching, drone detection and tracking, instantaneous velocity vector

## Abstract

This paper presents two techniques in the matching and re-identification of multiple aerial target detections from multiple electro-optical devices: 2-dimensional and 3-dimensional kinematics-based matching. The main advantage of these methods over traditional image-based methods is that no prior image-based training is required; instead, relatively simpler graph matching algorithms are used. The first 2-dimensional method relies solely on the kinematic and geometric projections of the detected targets onto the images captured by the various cameras. Matching and re-identification across frames were performed using a series of correlation-based methods. This method is suitable for all targets with distinct motion observed by the camera. The second 3-dimensional method relies on the change in the size of detected targets to estimate motion in the focal axis by constructing an instantaneous direction vector in 3D space that is independent of camera pose. Matching and re-identification were achieved by directly comparing these vectors across frames under a global coordinate system. Such a method is suitable for targets in near to medium range where changes in detection sizes may be observed. While no overlapping field of view requirements were explicitly imposed, it is necessary for the aerial target to be detected in both cameras before matching can be carried out. Preliminary flight tests were conducted using 2–3 drones at varying ranges, and the effectiveness of these techniques was tested and compared. Using these proposed techniques, an MOTA score of more than 80% was achieved.

## 1. Introduction

The detection of small objects through an electro-optical device is useful in augmenting manual surveillance techniques. The human eye is impervious to small and distant objects, which calls for the use of high-resolution cameras spread out across a wide area to detect such objects. With a network of cameras spanning a large surveillance space, it is important for cameras to identify common targets as they pass through each frame in order to maintain information about the objects from previous states. However, since each camera is limited to its own field of vision, matching algorithms must be employed to estimate the likelihood that two observed targets are the same physical object.

This paper formulates an inexact graph matching problem with the goal of maximizing the likelihood across the set of all possible pairwise matches, conditional on the positional and velocity data of all objects from the camera frames. Specifically, we aimed to find:

(1) $$\underset{I}{\mathrm{argmin}}\sum_{i\in S_{A}}\sum_{j\in S_{B}}I_{ij}\times l\left(i,j|X_{ij},V_{ij}\right)$$

where *I* is a vector with elements equal to one if *i* and *j* are selected for matching and *l* is the likelihood or probability that the match is correct, given the positional and velocity data *X* and *V*.

**Figure 1:**
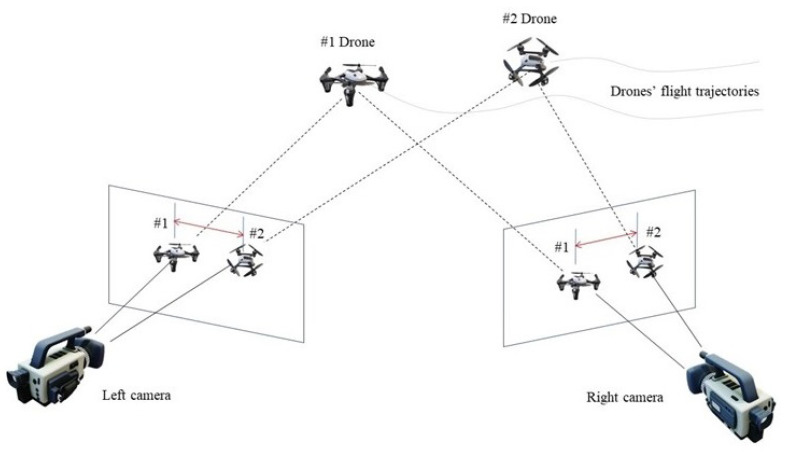
Schematic diagram of multiple cameras observing multiple targets for detection, tracking, and localization (2 cameras and 2 drones are shown). The multi-camera re-identification algorithm’s objective is to match the aerial targets that appear in different observing cameras and label them with the same IDs.

**Figure 2:**
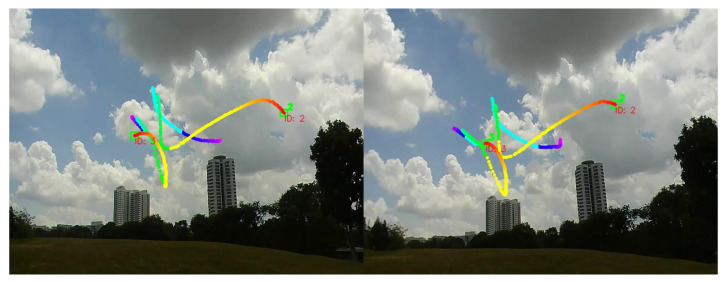
Example of matching two aerial targets that appear in 2 different observing cameras and labeling them with the same IDs of $ID_{2}$ and $ID_{3}$. Their trajectories are also shown, of which the blue color is the oldest and the red color is the latest.

Here, two distinct methods—two-dimensional and three-dimensional matching—are proposed. Both methods were applied to a specific case of using multiple electro-optical devices (cameras) to accurately track, locate, and re-identify the aerial targets entering the observation area, as shown in Figure 1 and Figure 2.

## 2. Literature Review

The Multi-Camera Multi-Target (MCMT) problem has been of significant interest in recent years, with prominent literature established by Ristani et al. [1,2]. One of the challenges in the MCMT problem involves target identification and re-identification, which is defined as the ability of an electro-optical device to detect an object, track its physical movement in a given region, and assign these tracks across cameras as the same target. Several applications in target re-identification include human motion analysis, robot navigation, event detection, anomaly detection, video conferencing, traffic analysis, and security. However, there remains a limited literature in the sub-domain of MCMT for aerial objects and drones. Moreover, existing research primarily focuses on on appearance- and image-based visual detection methods. Some examples include [3,4,5,6,7,8,9].

In 2011, Yang et al. [10] provided an in-depth study on the state-of-the-art progress of visual tracking methods. They explained the difficulties of visual tracking due to abrupt object trajectories, appearance pattern changes, non-rigid object structures, occlusions, and camera motion. Subsequently, Kulchandani [11] provided another review on moving object detection in 2015. Although both papers were methodologically detailed, there were no discussions on object matching or identification across camera platforms. Recently, Wang et al. (2017) [12] and Zhang et al. (2019) [13] presented refined methodologies to track and localize multiple targets from a single aerial camera mounted on a drone. Both separately provided comprehensive methods to correct visual constraints and for the motion of the viewing platform. However, their work was still based on a single camera and did not address target assignment across multiple cameras. Likewise, in 2020, Chapel and Boumans [14] provided a comprehensive paper on moving object detection, but with only a single moving camera and without extension to multiple cameras.

Prior to target re-identification, any relevant detection method may be used to generate targets. Specifically, motion-based target detection was adopted, using blob detection with target tracking through Kalman Filter-Discriminative Correlation Filter (KF-DCF) state-estimation, as delineated in Srigrarom et al. (2021) [15].

### Advantages of Kinematics-Based Techniques

This paper proposes and discusses in-depth the kinematics and motion-based techniques in performing target matching and re-identification. While there are several effective image-based target re-identification techniques [2,16,17], the major advantages of using motion-based over image-based techniques are:

1. There is no need to carry out image data training for target recognition in motion-based techniques;
2. There is no limitation to the camera pose as the algorithms are pose-independent;
3. These techniques are device invariant, with the possibility to integrate cameras operating at different frequency spectrums (e.g., normal vs. infrared cameras);
4. There is no ambiguity in repeated or similar targets, as long as the targets do not overlap or fuse together;
5. As long as the targets’ motions are traceable, re-identification can be performed. This creates an allowance for a certain level of noise in the obtained data (e.g., due to blurring, clutter, overlapping objects, shade, or occlusion).

## 3. Target Detection and Tracking

Before re-identification, a sufficiently robust detection method must be adopted. The aerial targets are captured in the monitored scene from multiple cameras. Image processing techniques are applied to remove background noise, by distinguishing between foreground and background sections. Morphological operations such as dilation and erosion are applied to remove noise and enhance signals. Next, thresholding and binarization of the frames are carried out to obtained masked frames. Finally, blob detection techniques are used to detect the targets.

The detected targets are further tracked by the combination of the Extended Kalman Filter (EKF) and Discriminative Correlation Filter (DCF) state estimation techniques. The EKF is used to initialize the detection, whereas the DCF is used for subsequent continuous tracking. The detections used for this paper were generated by a Discriminative Correlation Filter with Channel and Spatial Reliability (DCF-CSR), which is a novel approach to DCF tracking proposed by Lukežič et al. (2018) [18]. The detailed studies and selections were presented in a previous study (Seah et al., 2021) [19].

The Hungarian/Munkres algorithm is used to match detections to tracks. The matching algorithm is based on a two-dimensional cost matrix of tracks and detections, computed by comparing each detection’s Euclidean distance with the target state predictions from the DCF and KF state-estimation filters, as presented in Srigrarom et al., 2021 [15].

After the tracks are established in a multiple camera setup, it is possible to calculate the 3D position of each target by means of triangulation, as described in Srigrarom, et al., 2020 [20].

## 4. Two-Dimensional-Kinematics-Based Matching

This section proposes target re-identification using a 2-dimensional-kinematics-based matching method, based on several correlation forms of the targets’ motions extracted from the camera images.

Electro-optical devices such as cameras capture $I\;R^{3}$ objects as $I\;R^{2}$ projections. Due to this loss of dimensionality and the lack of positional and rotational information about the cameras, points of singularity may occur when trying to infer three-dimensional kinematic characteristics from two-dimensional information.

However, near-parallel optical cameras can be used to establish a matching algorithm between targets across frames, by relying on the geometric similarity between projected appearances to compute a confidence score between each pair of identified targets. To maximize the likelihood of target matches, the confidence scores rely on a total of three distinct geometric methods, which utilize both the $I\;R^{2}$ projected displacements and velocities of the identified targets.

### 4.1. Cross-Correlation of Normalized Velocity Vectors

While the projected velocity of the same target in two different cameras positioned at two different orientations will differ, it is likely that two cameras of near-parallel orientation will receive similar signals from the same target. Therefore, it is expected that the time series of velocity magnitudes will be strongly correlated across near-parallel projections of the same target.

For each target with a minimum history of *N* frames, we estimated the velocity of each target by computing the apparent change in position between consecutive frames. This vector of *N* velocities was then normalized to a mean zero and standard deviation one, and a discrete signals cross-correlation operation was performed, as in Equation (2).

(2) $$S_{1}\left(i,j\right)=\sum_{m}V_{i}\left[m\right]V_{j}\left[m+n\right]$$

### 4.2. Relative Position Matching

The relative position of identified targets in an $I\;R^{2}$ projection space is likely to be similar with minor aberration from the relative tilt of each camera. Hence, there is a high likelihood of a positive match if close neighbors of one target are geometrically similar to the close neighbors of another target in a different camera.

For each target, the relative Cartesian coordinates of each identified neighbor within a pre-defined radius *d* are calculated with respect to the target of interest. Then, a nearest-neighbors approach is used to pair each neighbor with its geometrically closest estimate. The resultant distance is compounded, and this total distance error is used as a loss metric with an inverse correspondence with the likelihood of matching.

(3) $$\frac{k}{S_{2}\left(i,j\right)}=\sum_{a\in S_{A}\backslash i}\min_{b\in S_{B}\backslash j}\left|X_{a}-X_{b}\right|$$

This method ultimately relies on the veracity of the other neighboring targets, which may not be perfectly or completely identified, and such must be used with the other two complementary methods for robust performance.

### 4.3. Directional Path History Matching

Lastly, we expected the projected turning angles of the same target in near-parallel cameras to be identical. Therefore, for each target with a minimum history of *N* frames, we constructed the perceived turning angle of each target as captured by each camera.

Between two sets of consecutive frame differences, the percentage change in heading with respect to one revolution is calculated and stored in an *N*-length vector and is adjusted such that the first velocity vector is pointing in a common direction across all cameras. A likelihood score is then computed using the average deviation of heading across all *N* observations.

(4) $$S_{3}\left(i,j\right)=1-\frac{1}{N}\sum_{t=1}^{N}\left|\theta_{i,t}-\theta_{j,t}\right|$$

### 4.4. Aggregate Likelihood Score

Based on the three likelihood scores created, we selected an appropriate set of weights $w_{1},w_{2},w_{3}$ to compute the overall likelihood score, l(i,j).

(5) $$l\left(i,j\right)=\sum_{k=1}^{3}w_{k}S_{k}$$

To solve the inexact graph matching problem, we therefore set up a complete bipartite graph with nodes representing the identified targets within each camera and weighted edges representing the likelihood *l* of a match between two targets *i* and *j*.

(6) $$L=\left[\begin{array}{ccccc} l(1,1) & l(1,2) & l(1,3) & ... & l(1,n)\\ l(2,1) & l(2,2) & l(2,3) & ... & l(2,n)\\ l(3,1) & l(3,2) & l(3,3) & ... & l(3,n)\\ ... & ... & ... & ... & ...\\ l(m,1) & l(m,2) & l(m,3) & ... & l(m,n) \end{array}\right]$$

The bipartite graph can be conceived of in the form of an adjacency matrix with individual elements of row *i* and column *j* corresponding to l(i,j). A recursive method such as the Hungarian or Munkres algorithm can be used to recursively identify global maxima subject to the criteria of $l\left(i,j\right)>l_{0}$ to prevent matches that are unlikely. The resultant set of matches form an estimate of the solution of the inexact graph matching problem.

### 4.5. Assumptions and Constraints

The effectiveness of this technique relies on the following assumptions and geometric constraints:

- The aerial targets must be observed and tracked by both cameras. The angle that subtends both focal axes is also restricted such that the small angle approximation is valid (i.e., near-parallel setup);
- The proposed re-identification techniques are based on the target motion and position. The data are also normalized to a range between 1 and −1. Therefore, the intrinsic and extrinsic parameters of the camera are not required;
- The cameras must be able to track the targets continuously. Although this method does not require image-based pattern recognition, the targets must be reasonably visible and not diminished by sub-pixel disappearances.

These assumption and constraints also apply to the 3D-kinematics-based matching, which is described in Section 5.

### 4.6. Field Tests

Two field tests were conducted to evaluate the performance of the 2D-kinematics-based matching algorithm. The setting and conditions were varied to ensure that the algorithm was location agnostic.

#### 4.6.1. First Field Test, Two Drones

Two small drones were manually flown in an open field independent of each other. The test flight was about 2 min long and may be reviewed at https://youtu.be/xiTd7On33Ds (accessed on 26 May 2021). Two cameras were set at a resolution of 1280 × 720 pixels, with a target frame rate of 30 frames per second. The cameras were connected to a central processing unit for to be fed into the algorithm.

**Figure 3:**
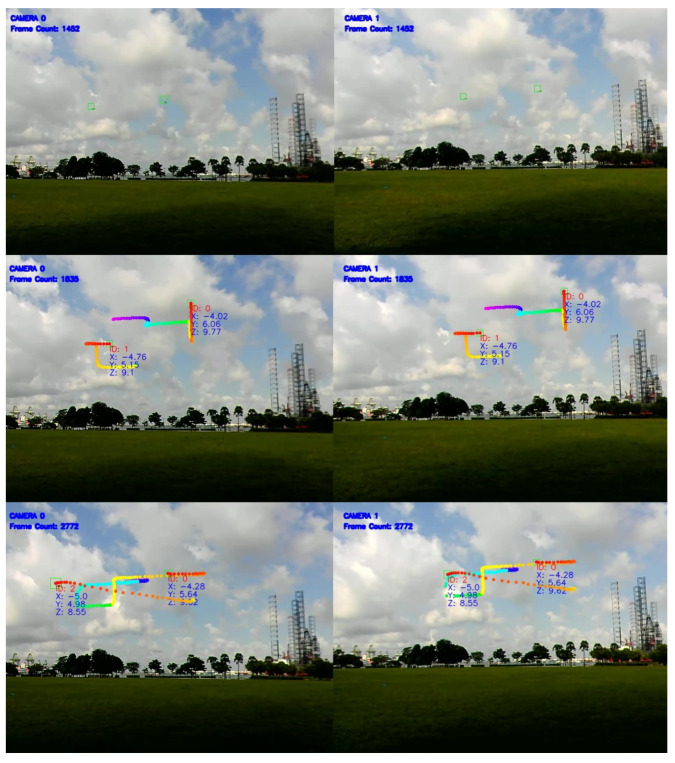
Still images from two ground cameras, with detected aerial targets (drones) successfully matched.

Figure 3 shows the still images of the re-identification process. In the first set of images in Figure 3, both drones were initially flown into the field of view of both cameras. Subsequent images show successful re-identification, with an identification assignment ($ID_{0}$) and ($ID_{1}$ or $ID_{2}$). It should be noted that one target may have multiple identification numbers across time due to intermittent detection by both cameras after a specified time period (more than 10 frames), as these targets are re-assigned as $ID_{2}$ upon re-detection and re-identification.

Overall, the re-identification algorithm achieved an Multi Object Tracking Accuracy (MOTA) of 90% averaged over each frame obtained in this field test, illustrating the effectiveness of the matching and re-identification capabilities across two cameras through the proposed 2D-kinematics-based matching algorithm. Further analysis of this result largely attributed the error to misdetections, which we can mitigate by using improved video resolutions. However, this requires hardware improvements to process larger image sizes to maintain a fixed frame rate.

#### 4.6.2. Second Field Test, Three Drones

A second test was performed using a higher number of aerial targets and with the same cameras and computer hardware as the first field test. The drones were similarly flown independently of one another in an open field. The test flight was approximately 2 min and may be reviewed at https://youtu.be/eAe-W3juIXw (accessed on 26 May 2021).

**Figure 4:**
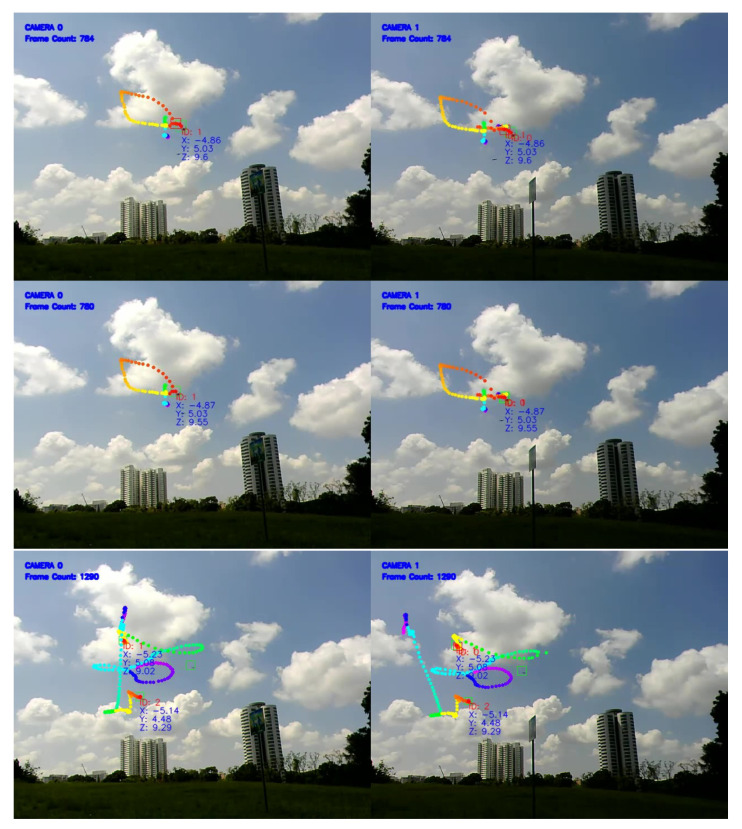
Still images from two ground cameras, with detected aerial targets (drones) successfully matched.

Likewise, Figure 4 demonstrates the successful re-identification with identification ($ID_{0}$), ($ID_{1}$), and ($ID_{2}$). Overall, this algorithm achieved an MOTA of 80% for this field test.

### 4.7. Discussion

The proposed multi-layer, two-dimensional kinematics-based matching algorithm ensures that mismatching between targets across two cameras can be minimized. Each layer is able to complement the strengths and weaknesses of other layers. For example, while drone formation flight scenarios might pose problems with only velocity- and turning-angle-based analysis (since all targets have a similar velocity vector and turning angles), the inclusion of relative positioning in the algorithm will assist in ensuring that each individual drone is matched to its correct counterpart across cameras.

The presence of unique noise tracks within frames may also cause inaccurate geometric shapes to be formed within each frame. While this may cause the second layer to be less accurate, the presence of velocity correlation and comparisons of the history of turning angles will remove noisy tracks, since these tracks by definition are stochastic in nature and do not converge to a predictable pattern. It is therefore important to include as many features as possible to identify corresponding tracks between both frames.

It is important to note however that the parameters set for this instance of the implementation were tuned or experimentally derived from test data. The efficiency of a successful match between two drone tracks is somewhat arbitrarily defined by the number of frames taken to achieve a successful match, and it is worthwhile to note that the algorithm is yet to be optimized such that the matching efficiency is maximized. A numerical analysis either by hand or by means of a learned model may be implemented in the future to achieve a higher matching efficiency with a sufficiently low false positive/negative rate.

Nevertheless, these results show that the system of multiple cameras can be a good low-cost position estimator for the tracking of drones in monitored 3D spaces. At present, the re-identification between the two cameras for aerial targets can achieve a high MOTA of >80% by frame count, as shown in the two flight tests. Furthermore, the current model is capable of re-identification of a lost track if the alternate camera still has a signature of the track.

However, when the drone track leaves both cameras’ frames completely and is out of the monitored space, a new track identification number is assigned to it if the same aerial track re-enters. The model is at present still unable to re-identify the same drone if it is lost from both cameras due to its trajectory-based matching system, which is independent of the target image.

## 5. Three-Dimensional-Kinematics-Based Matching

This section proposes target re-identification using a 3-dimensional-kinematics-based matching method. It involves a direct matching of targets based on a reconstructed instantaneous direction vector, which is in contrast to the previous method that uses multiple correlations instead of direct matching.

The central idea involves the reconstruction of a three-dimensional orientation vector in the global frame using changes in the pixel coordinate and target size to be used for matching across independently oriented cameras. As the information available from an $I\;R^{2}$ projection is insufficient to reconstruct a point in $I\;R^{3}$, we can estimate the third dimension of depth by using changes in the size of the target in the 2D projection. When the object is sufficiently near, the classical lens equation may be applied.

(7) $$\frac{1}{d}+\frac{1}{i}=\frac{1}{f}$$

where *d* is the physical object distance in the third dimension (depth) from the lens, *i* is the image distance in the third dimension from the lens, and *f* is the focal length of the lens. The linear magnification is:

(8) $$M=\frac{-i}{d}=\frac{h}{H}$$

where *M* is the magnification. *h* is the image size, and *H* is the object size, which is position invariant. If the same camera is used, *M* and *f* are fixed. If the object moves across time, the difference in the image size, i.e., $h_{1},h_{2}$, can be measured to derive the relationship of object distances, $d_{1},d_{2}$:

(9) $$d_{1}-d_{2}=fH\left\{\frac{1}{h_{1}}-\frac{1}{h_{2}}\right\}$$

With $d_{1},d_{2}$, the objects’ velocity can be estimated in the third dimension (depth).

The area ratio *r*, object displacement Δx and Δy, is introduced as follows:

(10) $$r=\sqrt{\frac{A_{t}}{A_{t-k}}}$$

where *k* is the number of frames delayed. To attain noticeable differences in motion to compute the area ratio, *r*, frames of at least 0.5 seconds apart are compared. This corresponds to a comparison of the images at frames *t* and (*t* − 15) for a 30 FPS video with *k* = 15. The use of delayed frames over consecutive frames assists in minimizing noise arising due to detection capabilities.

We further show that the object displacement Δx and Δy and the subsequent velocity direction vector are independent of depth *d*. Let the plane *S* be normal to the camera lens and containing the velocity vector *v*. The field of view angle is $FOV_{x}$. The total number of pixels in the *x* and *y* directions is $P_{x}$ and $P_{y}$, respectively. Let *r* be the area ratio such that if the initial depth is *d*, the corresponding final depth is d/r. The corresponding object’s movement in the *z* direction (depth) is:

(11) d/r−d=d(1/r−1)

(12) $$d_{1}-d_{2}=fH\left\{\frac{1}{h_{1}}-\frac{1}{h_{2}}\right\}$$

**Figure 5:**
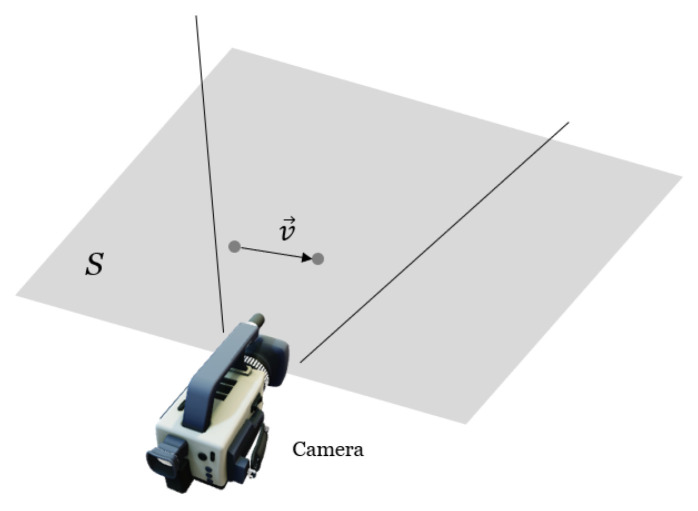
Three-dimensional view of the camera setup.

**Figure 6:**
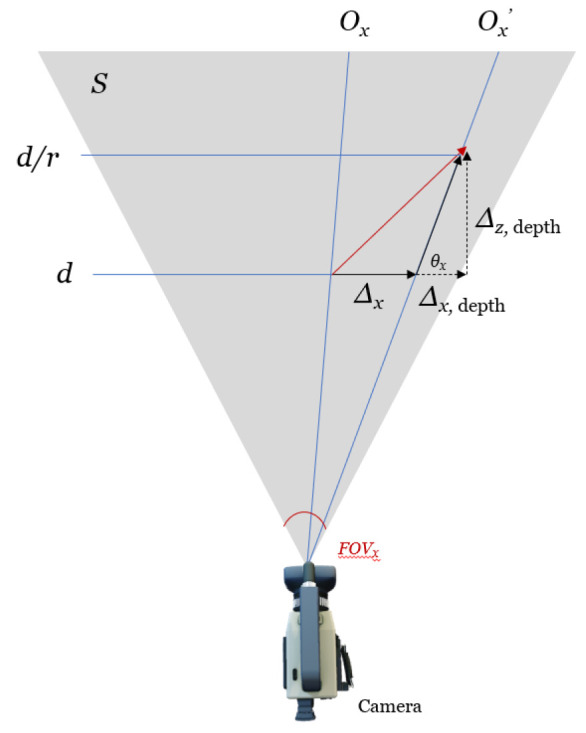
Two-dimensional schematic diagram.

A plan view of the plane *S* is simulated in Figure 5 and Figure 6.

**Figure 7:**
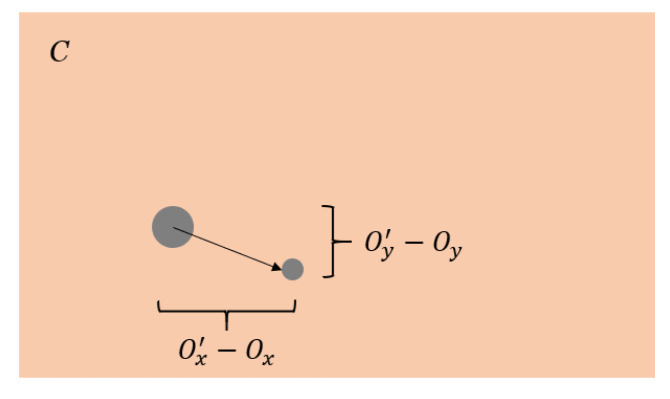
Object in the camera frame.

For the objects in the camera frame, we can measure:

1. Pixel distance $\left(O_{x}^{\prime }-O_{x},O_{y}^{\prime }-O_{y}\right)$, as shown in Figure 7;
2. Blob area ratio (r) between frame;
3. Field of view FOV in the *x* and *y* directions.

We observe the centroid of the target, $O=(O_{x},O_{y})$ and $O^{\prime }=\left(O_{x}^{\prime },O_{y}^{\prime }\right)$.

(13) $$\Delta x=\frac{\left(O_{x}^{\prime }-O_{x}\right)}{P_{x}}\times 2d\tan\left(\frac{FOV_{x}}{2}\right)$$

(14) $$\Delta x,depth=\frac{d\left(\frac{1}{r}-1\right)}{\tan\theta_{x}}$$

(15) $$\Delta x,total=\frac{\left(O_{x}^{\prime }-O_{x}\right)}{P_{x}}\times 2d\tan\left(\frac{FOV_{x}}{2}\right)+\frac{d\left(\frac{1}{r}-1\right)}{\tan\theta_{x}}$$

(16) $$\Delta z=d\left(\frac{1}{r}-1\right)$$

where $\theta_{x}=\frac{\pi }{2}-\left\{O_{x}^{\prime }-\frac{P_{x}}{2}\right\}\frac{FOV_{x}}{P_{x}}$. We define the displacement in the y-axis similarly so that:

(17) $$\Delta y,total=\frac{\left(O_{y}^{\prime }-O_{y}\right)}{P_{y}}\times 2d\tan\left(\frac{FOV_{y}}{2}\right)+\frac{d\left(\frac{1}{r}-1\right)}{\tan\theta_{y}}$$

where: $\theta_{x}=\frac{\pi }{2}-\left\{O_{x}^{\prime }-\frac{P_{x}}{2}\right\}\frac{FOV_{x}}{P_{x}}$ Then, from (7)–(9), we have:

(18) $$\left[\begin{array}{c} \begin{array}{c} \Delta x\\ \Delta y\\ \Delta z \end{array} \end{array}\right]=d\left(\begin{array}{c} \begin{array}{c} \frac{\left(O_{x}^{\prime }-O_{x}\right)}{P_{x}}\times 2\tan\left(\frac{FOV_{x}}{2}\right)+\frac{\left(\frac{1}{r}-1\right)}{\tan\theta_{x}}\\ \frac{\left(O_{y}^{\prime }-O_{y}\right)}{P_{y}}\times 2\tan\left(\frac{FOV_{y}}{2}\right)+\frac{\left(\frac{1}{r}-1\right)}{\tan\theta_{y}}\\ \left(\frac{1}{r}-1\right) \end{array} \end{array}\right)$$

which proves that the instantaneous velocity vector is independent of depth *d*.

It is also observed that with the addition of focal length *f* and the object size *H*, through (3) and (8) such that $\Delta z=d_{1}-d_{2}$, the absolute depth *d* may be separately calculated.

The Δx, Δy, and Δz calculated may be used for matching across cameras if the poses of these cameras are known, such that a series of coordinate transformations using Euler angles and rotation matrices may be performed from the local frame to the global frame of reference. The vector was normalized to one for comparison and matching among observing cameras. As the calculated vector from each camera is independent of pose, a direct matching using correlation can be performed.

(19) $$\left[\begin{array}{c} \begin{array}{c} {\hat{V}}_{x}\\ {\hat{V}}_{y}\\ {\hat{V}}_{z} \end{array} \end{array}\right]=\left[\begin{array}{c} \begin{array}{c} V_{x}\\ V_{y}\\ V_{z} \end{array} \end{array}\right]/\sqrt{V_{x}^{2}+V_{y}^{2}+V_{z}^{2}}$$

### 5.1. Field Tests

**Figure 8:**
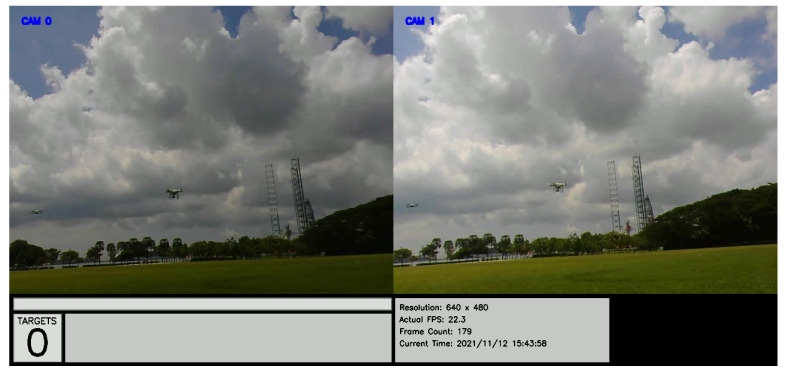
Still images from the two ground cameras, showing a snapshot of the drones flying in an open field.

Similar to the previous method, field tests were conducted using multiple drones flown manually and independently in an open field. The test results may be reviewed at https://youtu.be/JoinccrL7b8 (accessed on 14 November 2021). Still images of the test are shown in Figure 8. An overall MOTA of 77% was obtained for this test.

**Figure 9:**
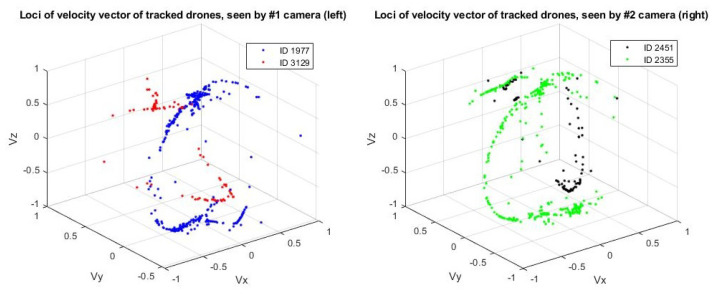
Loci of the velocity of the tracked drones from both the left and right cameras. ID 1977 from the left camera matches with ID 2355 from the right camera and is assigned ($ID_{1}$). Likewise, ID 3129 from the left camera matches with ID 2451 from the right camera and is assigned ($ID_{0}$).

**Figure 10:**
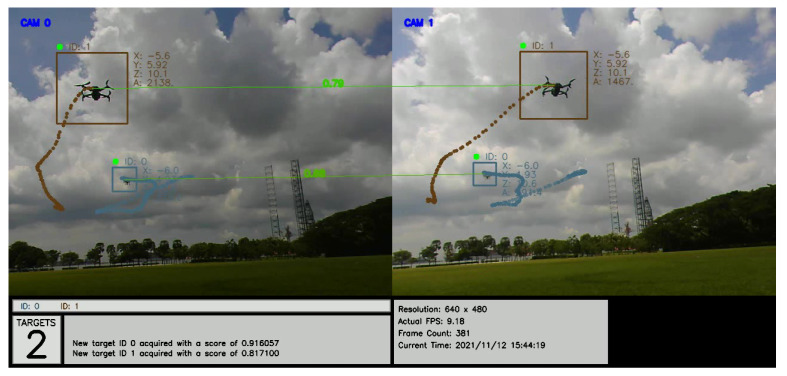
Images from the two ground cameras, showing a snapshot of the drones detected, tracked, and labeled with IDs with their 3D positions (x, y, z). The connecting lines show positive matching between identified targets.

Figure 9 shows a plot of the direction vectors for each target over time. In this sample result, ID 1977 from the left camera matches with ID 2355 from the right camera and is re-identified successfully as ($ID_{1}$). Likewise, ID 3129 from the left camera matches with ID 2451 from the right camera. The algorithm is also able to match both of them (ID 3129 and ID 2451) and re-identify them as ($ID_{0}$), as shown in Figure 10.

**Figure 11:**
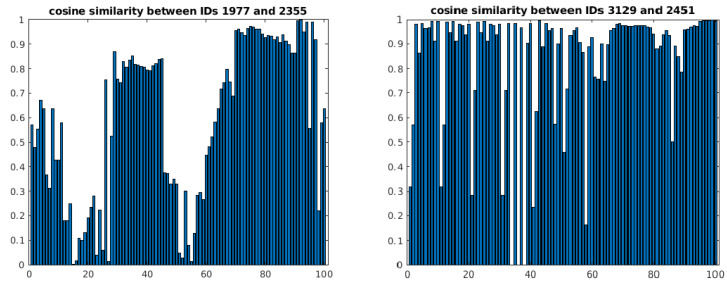
Interpolated cosine similarity scores of matched velocity vectors with IDs 1977 and 2355 (**left**) and IDs 3129 and 2451 (**right**). A higher cosine similarity score means that the vectors are more identical to each other.

To have a better idea of the measurement errors in the matching process, the interpolated cosine similarities of the matched velocity vectors from Figure 9 are plotted. The more identical two vectors are, the higher their cosine similarity score (perfectly identical vectors have a score of one) [21]. As the individual points of the matched tracks in Figure 9 do not always correspond to the same timestamps, a point-by-point comparison of the cosine similarities was not possible. Instead, interpolation of the matched tracks was carried out and used to approximate the similarities of the matched tracks over time. The results are shown in Figure 11.

We can observe that the average cosine similarities of both pairs of matched tracks are high, but do not converge to one. This shows that the 3D-kinematics-based method does not necessarily require the generated velocity vectors to be perfectly identical to each other for matching to take place and, instead, can operate on an inexact matching principle.

**Figure 12:**
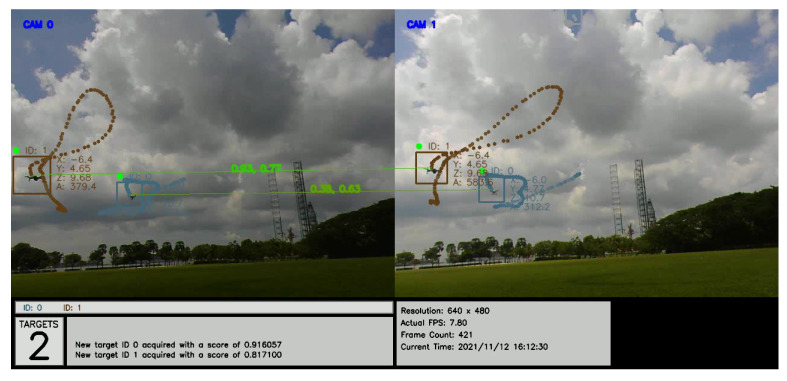
Images from the two ground cameras, showing a snapshot of the drones detected, tracked, and labeled with IDs with their 3D positions (x,y,z). The connecting lines show positive matching between identified targets. The first and second scores correspond to the 2D- and 3D-kinematics-based methods respectively.

An annotated recording of the test with comparative matching scores may be viewed at https://youtu.be/QoZwQgUjZxU (accessed on 15 November 2021) and is also shown in Figure 12. The first and second scores correspond to the 2D- and 3D-kinematics-based methods, respectively.

### 5.2. Discussion

The key advantages for the three-dimensional-kinematics-based matching are as follows:

1. This method is able to estimate a three-dimensional motion vector from two-dimensional information;
2. As the 3D velocity vector is created and tracked, any fluctuations or noise in 1D or 2D are significantly minimized;
3. This technique is suitable for objects in the near to medium range where the object size is noticeable. This can also be improved by increasing the number of pixels, such as with the use of Ultra-High-Density (UHD) cameras.

The flight test results demonstrated that both re-identification techniques are sufficiently robust as long as continuous detection and tracking of the targets can be maintained. The 2D technique appears to provide better matching scores for targets that are far away as compared to the 3D technique. However, when changes in target sizes are significant, the 3D-kinematics-based method provides better matching scores. This implies that the targets must sufficiently near to the camera lens for the 3D technique to be effective, whereas the 2D technique can be applied to variable ranges as long as the targets’ motions can be observed. A combination of both 2D and 3D techniques will ensure effective matching and re-identification by integrating the strengths of both methods.

While there are still interruptions in re-identification, these can be attributed to the limitations of the detection algorithms rather than the re-identification techniques. Examples of interruptions include occlusion when targets cross behind obstacles, or overlapping when two targets cross paths. In the flight test scenarios where the aerial targets are flown in a clear sky with minimal occlusion or overlap, continuous detection and tracking of the targets can be maintained, which allows the re-identification techniques to work effectively.

## 6. Conclusions and Future Work

This paper presents the re-identification of multiple aerial tracked targets from multiple cameras through two proposed kinematics-based methods: two-dimensional and three-dimensional kinematics-based matching. The main advantages of these methods over traditional image-based methods are: (1) no prior image recognition training is required; instead, graph matching algorithms, which are relatively simple to deploy, are used; (2) one can use various types of cameras for more operational flexibility; (3) the targets can be similar and/or repeated; (4) there is an allowance for errors such as blurred, overlapped, occluded, or shaded conditions, as long as the moving targets can be tracked.

The 2D-kinematics-based technique is based on multiple-layer correlations of targets’ motions and can be used for multi-range (near to far) scenarios as long as the targets can be detected and tracked. The 3D-kinematics-based technique uses the estimation of the depth motion and the construction of the 3D instantaneous velocity vector for direct matching. The 3D technique provides better matching scores when the targets are near to medium range where changes in target sizes are observable. When used together, both techniques provide robust matching of targets from near to far range by complementing the strengths and weaknesses of each other.

Initial proof-of-concept outdoor field tests were conducted and showed that both techniques were able to re-identify all drones correctly as long as the targets are detected and tracked. On average, an MOTA score of approximately 80% across cameras for both techniques was achieved. Any interruptions or mismatches appeared to be issues arising from the limitations of the detection algorithms.

The next step of our research will be to expand the algorithm to account for more diverse scenarios, such as having to detect targets in the presence of increased noise and occlusion (e.g., targets flying along the treeline instead of in the sky) or increasing the quantity of tracked targets while taking into account limited processing time and computing resources.

## Data Availability

Not applicable.
